# The Plague and Anti-Toxic Serum

**Published:** 1897-09-18

**Authors:** 


### THE PLAGUE AND ANTI-TOXIC SERUM.

Of the various speakers at the Moscow Congress none, perhaps, received a greater ovation than Professor Metchnikoff, of Paris. Speaking of the Plague and of its treatment by anti-toxin, he explained why the serum tried in India had proved so much less effective than that which had been used by China. The reason was that because of the fear of causing the spread of the disease in Europe, the thirty horses inoculated in Paris to provide anti-toxic serum for use in India were inoculated with dead cultures, whereas the serum which had been used by Yersin in China was obtained after inoculation with living cultures. Thus the mortality of the Chinese cases in which the serum was employed was only 7 per cent., while that of the Indian cases was 49 per cent. Nevertheless, poor as it was, the serum was better than nothing, the natural mortality of plague when untreated being 80 per cent. The inoculations produced absolutely no reaction, and their protective influence was as lasting as that produced by Haffkine's method. A general employment of this protective serum would, he said, guarantee the civilised world against an invasion of the plague. We may suggest, however, that the civilised world is not yet convinced that, even without inoculation, plague is any serious danger to civilised, or, in other words, to cleanly communities.

